# Minocycline and Magnesium As Neuroprotective Agents for Ischemic Stroke: A Systematic Review

**DOI:** 10.7759/cureus.12339

**Published:** 2020-12-28

**Authors:** Juan Fernando Ortiz, Samir Ruxmohan, Alisha Saxena, Álvaro Morillo Cox, Farah Bashir, Willians Tambo, Mohammad R Ghani, Gustavo Moya, Ignacio Córdova

**Affiliations:** 1 Neurology, Universidad San Francisco de Quito, Quito, ECU; 2 Neurology, California Institute of Behavioral Neurosciences & Psychology, Fairfield, USA; 3 Neurology, Larkin Community Hospital, Miami, USA; 4 Neurology, Dayanand Medical College and Hospital, Ludhiana, IND; 5 Medicine, Universidad San Francisco de Quito, Quito, ECU; 6 Internal Medicine, Liaquat University of Medical & Health Sciences, Hyderabad, PAK; 7 Emergency Medicine, Carlos Andrade Marín Hospital, Quito, ECU; 8 Medicine, Universidad Internacional del Ecuador, Quito, ECU

**Keywords:** stroke, minocycline, magnesium

## Abstract

Stroke is a leading cause of death, disability, and dementia worldwide. Strokes can be divided into ischemic strokes and hemorrhagic strokes. At the moment, tissue plasminogen activator (tPA) is the only FDA-approved drug for ischemic stroke. Minocycline (MC) and Magnesium (Mg) are promising therapies for ischemic stroke, especially in the pre-hospital setting. These drugs are readily available, inexpensive, and generally safe. We decided to investigate these drugs' neuroprotective effects in treating ischemic stroke in the acute and chronic setting. We conducted a systematic review of the published literature on MC and Mg's functional outcome in ischemic stroke. This paper's methodology included only clinical trials published in the last 15 years, using PubMed as a database. The systematic review demonstrated that MC infusion in the pre-hospital and hospital setting improved functional outcomes and disability scores.

Furthermore, MC also decreased matrix metalloproteinase 9 (MMP-9) levels. MC might have a more significant effect on men than women because different molecular pathways of cerebral ischemia seem to be involved between both genders. The systematic review showed that patients with ischemic stroke did not benefit from magnesium sulfate infusion in the pre-hospital and hospital setting. Nevertheless, patients with lacunar strokes and patients who supplemented their meals with potassium-magnesium salt in the diet had better functional outcomes. Future studies would need a more significant sample of participants and a better selection to increase the study's power and avoid selection bias, respectively. Further publications could benefit from subcategorizing strokes and investigating the gender role in stroke treatment. These directives could give a more robust conclusion regarding the neuroprotective effects of these drugs.

## Introduction and background

Stroke is the third leading cause of death in adults, the second most leading cause of dementia, and the most common cause of disability globally [1]. There is an ongoing search for treatments that can reduce the mortality and disability associated with stroke. At the moment, only tissue plasminogen activator (tPA) and antiplatelets are approved drugs for acute treatment. Another important fact is that only 2-3% of patients with ischemic stroke receive tPA in the United States; due to tPA's short therapeutic window of 4.5 hours and the increased risk of hemorrhagic conversion [2]. Thus, there is a need to investigate new therapies for ischemic stroke.

Cerebral ischemia causes the activation of intracellular and extracellular proteolytic cascades, leading to a disruption of the Blood-Brain Barrier (BBB). Interruption of this process could be beneficial for the treatment of ischemic stroke. Minocycline (MC) is a semi-synthetic tetracycline antibiotic that has been to shown neuroprotective effects. The drug is lipophilic, which allows the drug to cross the blood-brain barrier [3]. MC is increasingly being considered a neuroprotective drug due to its high penetration into the BBB. Minocycline also showed clinical improvement in various neurodegenerative diseases [4]. Magnesium (Mg) has shown in preclinical studies to have neuroprotective effects. It also has anti-inflammatory and anti-apoptotic abilities [5]. Mg increases regional cerebral blood flow to ischemic brain areas, enhancing cellular metabolism recovery. Mg is usually administered as intravenous (IV) magnesium sulfate in clinical trials [6].

Magnesium sulfate and minocycline are inexpensive, safe, and easily administrated [5,7]. Combining these drugs in the pre-hospital setting could be an excellent strategy to improve patients with ischemic stroke in remote areas and countries where stroke unit care or tPA is unavailable [7]. Our objective is to review these drugs' clinical trials and analyze if it is a good strategy to use one or both therapies in the pre-hospital setting. We will discuss the pathophysiology, the limitations and the overall efficacy of the clinical trials in this systematic review.

## Review

Methods

Protocol

We conducted a systematic review in compliance with the Preferred Reporting Items for Systematic Reviews and Meta-Analysis (PRISMA) [8]

Eligibility Criteria and Study Selection

We included only the clinical trials conducted in the last 15 years in the English language. We excluded all animal studies, studies other than clinical trials, and papers that did not fulfil the study's outcome. Patients in the study also needed to be more than 18 years old. After this process, we remove duplicate papers and studies in which the title was not pertinent. 

After screening the studies, we included only papers that have one of the following characteristics:

1. Patients: individuals with ischemic stroke

2. Intervention: Mg or MC treatment in patients with ischemic stroke.

3. Comparator: placebo or control group.

4. Outcomes: one of the following modified Rankin scale (mRS), National Institute of Health Stroke Scale (NIHSS), Barthel Index (BI), and overall mortality.

Database and Search Strategy

For this systematic review, we used PubMed as a database to search the studies for our systematic review. The search was done between 11/28/2020- 12/02/2020. The combination of search terms that we used was "stroke" and "minocycline" & "stroke" and "magnesium" OR "magnesium sulfate. " We did a quick search of Medline and Scopus, and we did not find any other clinical trial different from the ones in PubMed. That is why our flowchart only shows the information gathered in PubMed.

Data Extraction

We collected the information that included: author & year of publication, methodology, and functional outcomes. Baseline characteristics of the study methods include the number of participants in the treatment, number of participants in the control group, dose route of administration of the drugs, duration of treatment, and timing when the drugs were giving base on the onset of symptoms. Baseline functional outcomes included mRS and NIHSS scores, BI, and overall mortality. Table 1 summarizes the methods used in this systematic review.

**Table 1:** Methodology of the systematic review in this paper

Key Terms	Database	Before Inclusion/exclusion criteria (results)	After Inclusion/exclusion criteria (results)	After the screening process
"Stroke” AND "Minocycline"	PubMed	208	11	5
"Stroke” AND “Magnesium” OR “Magnesium Sulfate”	PubMed	742	40	4

Bias Assessment

We have used the Cochrane collaboration's risk of bias tool to assess the risk of bias in the studies' clinical trials [9].

Results

**Figure 1:**
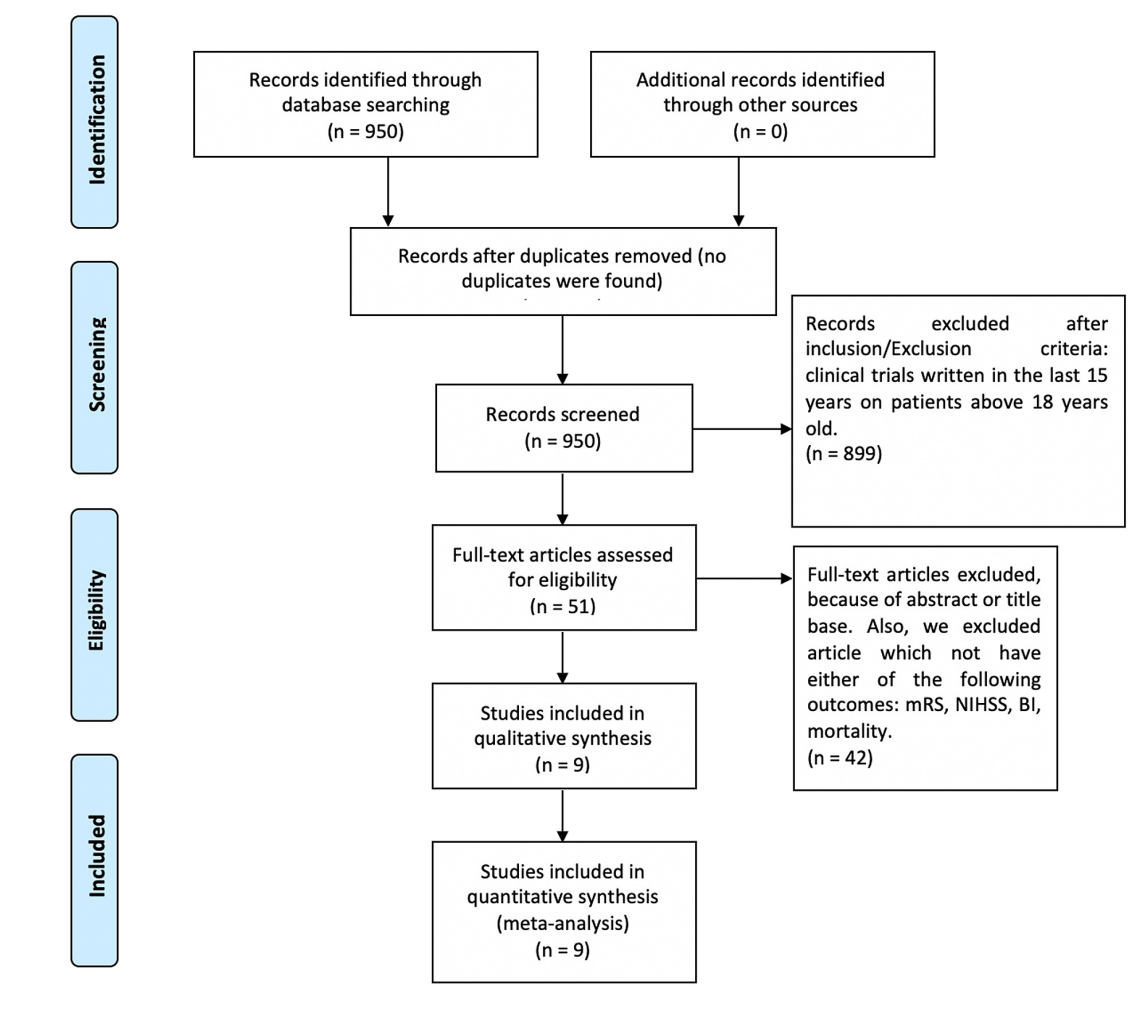
Flowchart of the systematic review mRS: modified ranking scale, mBI: Barthel Index, NIHSS: National Institute of Health Stroke Scale

Figure 1 shows a flowchart with the results of this systematic review.

Study Characteristics

We found five clinical trials of minocycline for the treatment of ischemic stroke. The oldest article was conducted in 2007, and the latest article was published in 2014. The clinical trials were conducted in three different countries. Table 2 showed the study characteristics of the minocycline clinical trials [3,4,7,10,11].

**Table 2:** Study characteristics of the clinical trials of minocycline on ischemic stroke. tPA: tissue plasminogen activator, mg: miligrams, IV: intravenous

Study, year of publication	Drug	Country	Study Design	Number of patients in Treatment group	Number of patients Control	Dose, duration, route, and onset of symptoms
Switzer et al. (2011) [10]	Minocycline	United States	This was a phase 3, multicenter, non-randomized, double-blind, placebo-control trial. A separate cohort that did not receive minocycline was used.	60	44	Four doses (3.0, 4.5, 6.0, 10.0 mg/kg), over one hour every 12 hours for three days, IV, within six hours of symptom onset If the subject received tPA, the baseline sample was taken at the moment of tPA administration.
Amiri-Nikpour et al. (2014) [4]	Minocycline	Iran	Open-label evaluator-blinded trial	26	27	200 mg, once daily for five days, oral route, 6-24 hours from the onset of symptoms.
Kohler et (2013) al. [7]	Minocycline	Australia	This was a clinical trial, multicenter prospective randomized open-label blinded end point	47	48	100 mg, 12 hours for five doses, IV, within 24 hours of onset symptoms
Padma Srivastava et al. (2012)[10]	Minocycline	India	Randomized single-blinded open-label study	23	27	200 mg/day for five days, oral, 6-24 of the onset of symptoms The control group received oral vitamin B capsules.
Lampl et al. (2007) [11]	Minocycline	United States	Randomized, open- label, evaluator-blinded clinical trial.	74	77	200 mg, for five days, oral, within 6-24 of onset of symptoms.

We found four clinical trials of magnesium for the treatment of ischemic stroke. The oldest article was conducted in 2007, and the latest article was published in 2017. The clinical trials were conducted in three different countries. Table 3 show the study characteristics of the clinical trials of magnesium [5,6,12,13].

**Table 3:** Study characteristics of the clinical trials of magnesium on ischemic stroke. g: grams, Mg: magnesium, IV: intravenous, ml: milliliters,

Study, year of publication	Drug	Country	Study design	Number of patients in Treatment group	Number of patients Control	Dose, route and duration, onset of symptoms
Aslanyan et al. (2007) [5]	Magnesium- Sulfate	United Kingdom	Post-hoc analysis of a randomized, multicenter trial Hemorrhagic stroke was excluded. There was the inclusion of only two of the classic lacunar syndromes (pure motor and sensorimotor strokes)	383	382	IV, continued for 24 hours within 12 hours of the onset of stroke, continued for 24 hours
Pan et al. (2017) [6]	Potassium-magensium salt	Taiwan	Double blind, multicenter, randomized control trial The patients were divided into three groups: Regular salt, potassium enriched salt and magnesium potassium enriched salt.	Potassium - magnesium-enriched salt (n = 95)	(n = 99) (regular salt) (Na/Cl salt) (n= 97) (potassium enriched salt) (K salt)	4.1 mmol for magnesium and 44.8 mmol for potassium, oral, for six months
Shkirkova et al. (2017) 12]	Magnesium-Sulfate	United States	Randomized, blinded and placebo-controlled trial Patients were divided into five quintiles based on the blood Mg levels.	569	561	15-minute loading dose, infusion then, 4g magnesium-sulfate or placebo followed by a 24-hour maintenance dose of 16 g magnesium-sulfate or matched placebo over 24 hours, IV, within 24 hours of the onset of symptoms
Saver et al. (2015) [13]	Magnesium-Sulfate	United States	Phase 3, multicenter, randomized, double-blind, placebo-controlled, pivotal clinical trial.	855	840	Bolus (loading) dose contained 4 g of magnesium sulfate in 54 ml of normal saline infused over a period of 15 minutes; the maintenance infusion contained 16 g of magnesium sulfate diluted in 240 ml of 0.9% normal saline, infused at a rate of 10 ml per hour for 24 hours, IV, within two hours of symptoms

We compared the following functional outcomes in the clinical trials: NIHSS, mRS, BI, and mortality. NIHSS measures the deficits caused by a stroke. The scale ranges from 0 to a maximum of 42, subdivided into 11 evaluated categories [14]. The mRS (Grade 0-6) measures patients' degree of disability after suffering a stroke. The mRS is the most common scale used in clinical studies [15]. The scale requires prospective data and is vulnerable to attrition bias. The scale goes from one-six. The higher the number, the more disability exist [15].

The BI measures the levels of disability and the patients' capacity to develop daily activities. The system has a score from 0-100. It has 10 items, and unlike the other scales, the higher the number of the scale, the better the patient's outcome [16]. Table 4 summarizes the outcomes and the conclusions of the clinical trials [3-7,10,12,13].

**Table 4:** Results and conclusions of the clinical trial of Minocycline and Magnesium mRS: modified ranking scale, mBI: Barthel Index, NIHSS: National Institute of Health Stroke Scale, N/A: not applicable or not described, Na: sodium, K: potassium, IV: Intravenous, g: grams, MRI: magnetic resonance imaging; GCS: Glasgow Coma Scale

Author, Year of Publication	Outcomes	Conclusions
Switzer et al. (2011) [10]	NIHSS, MMP-9 levels	MMP-9 levels were lower at 72 hours comparing with a baseline for both tPA, and non tPA treated subjects with statistically significant results. The tPA group had statistically significantly lower levels at one hour and 24 hours. The NIHSS was lower after seven days in patients taking minocycline.
Amiri-Nikpour et al. (2014) [4]	NIHSS	NIHSS score was significantly lower in the minocycline-treated group compared with controls. Female participants did not have significant clinical improvement measured by NIHSS compared with males. Patients who received oral minocycline daily for five days had better neurological outcomes on days 30, 60, and 90 compared to controls.
Kohler et al. (2013) [7]	mRS, NIHSS, mortality	Intravenous minocycline was safe but did not reduce death or dependency after 90 days or improve any functional outcome (mRS, NIHSS).
Padma Srivastava et al. (2012) [10]	NIHSS, mBI, mRS, MRI characteristics, mortality	mRS was significantly lower on day 90, NIHSS was significantly lower on day 30 and 90 in the treatment group; mBI scores were significantly higher in the treatment group. MRI T2 DWB imaging did not show a significant difference in the lesion volume between both groups. There was no difference in mortality.
Lampl et al. (2007) [11]	NIHSS, BI, mRS, mortality	NIHSS and mRS were significantly lower. At the same time, BI scores were significantly higher in the treatment group. This pattern was apparent since day 7 and maintained on day 30. There was no difference in mortality or hemorrhagic transformation between the two groups.
Aslanyan et al. (2007) [5]	BI, mRS, Glasgow Outcome Score (GOS), mortality	Younger patients, patients with higher baseline diastolic blood pressure, higher mean blood pressure, and absence of ischemic heart disease showed statistically significantly improved results. They showcased the beneficial effect of Mg treatment. Mg improved Barthel index<95, modified Rankin scale>1, and global outcome but not Barthel Index <60. There was no difference in mortality between the two groups.
Pan et al. (2017) [6]	NIHSS, mRS, BI	Patients taking dietary K/Mg salt had greater neurological performance in the NIHSS, MRS, and BI scales after six months.
Shkirkova et al. (2017) [12]	mRS, NIHSS, BI, mortality, GCS	The gravity-controlled method help patient to rapidly achieve steady levels of Mg. Nevertheless, there was no relation between Mg levels and clinical outcomes. Patients with the highest quintiles of Mg level did not modify the day 90 functional outcome (mRS 0-1), 90-day efficacy outcomes of Barthel index>90, NIHSS<1. GCS did not differ between the two groups. Mortality did not differ by quintiles.

Limitations of the Clinical Trials of Minocycline and Magnesium

The study by Switzer et al. was not a randomized controlled trial and lacked a proper control group. To analyze the bias in this study, we use the ROBINS-I tool, for the rest of the studies, we use the Cochrane collaboration's risk of bias tool [17,9]. In this study, the comparison group was from a separate cohort that did not receive minocycline. There was selection bias due to an imbalance among the predisposing conditions and race. At the same time, the sample was too small [10]. Table 5 summarizes the bias assessment of Switzer's study [10].

**Table 5:** Bias assessment of Switzer study using ROBIN-I tool for non-randomized clinical trials

Study	Confounding	Selection bias	Classification of intervention	Deviation from Intervention	Missing data	Measurement of the outcome	Selection of reported result
Switzer et al. (2011) [10]	Low	Moderate	Low risk	Low risk	Moderate	Low risk	Low risk

The study of Amiri-Nikpour et al. also demonstrated positive results but only in men. While it is plausible that minocycline only has a therapeutic effect on men, it is also important to note that the sample size was small. The administration route was oral, and the author points out that future studies should investigate if the oral or IV administration has more efficacy in patients [4].

The study conducted by Kohler et al. failed to show clinical improvement in patients taking minocycline. There was a lack of statistical power in the study. The lack of efficacy may be a false-negative (type II error). This result means that minocycline could potentially improve survival free of handicap. A small number of participants was the primary account for the study's lack of power [7].

The study by Padma Srivastava et al. also had a small sample. Other limitations included the study's unblinded nature, an undefined subtype of stroke, the route of administration was oral instead of IV. Mg levels can interact with minocycline. Mg levels were not monitored, which could result in inaccurate results. Also, the time frame for the onset of symptoms was 6-24 hours. Maybe early intervention could generate better outcomes [3].

Finally, in the Lamp et al. trial, there were some limitations as design (open-label, evaluator-blinded, confirmed by a double-blinded, uncontrolled), the dosage of minocycline, route of drug administration, the small size of the sample, and the time of drug administration (at six and 24 hours after stroke [11].

In the United States, 91-94% of patients have ischemic strokes, while 6% - 9% have hemorrhagic strokes [18]. In the study of Saver et al. there was a higher enrollment of patients with a hemorrhagic stroke, which does not represent the reality in the United States [5]. One explanation was that patients with hemorrhagic strokes tend to be more symptomatic and faster to seek medical care. The same study points out that the length of the study was eight years. While treatment has not changed in eight years, the delivery of therapies has changed during that time [5].

The study of Aslanyan et al. demonstrates that patients with lacunar stroke had better clinical outcomes. However, this was a post hoc analysis and should be confirmed with an RCT [12]. The study by Shkirkova et al. was very well conducted. We determined that the study had a small risk of bias in all domains [13]

**Figure 2:**
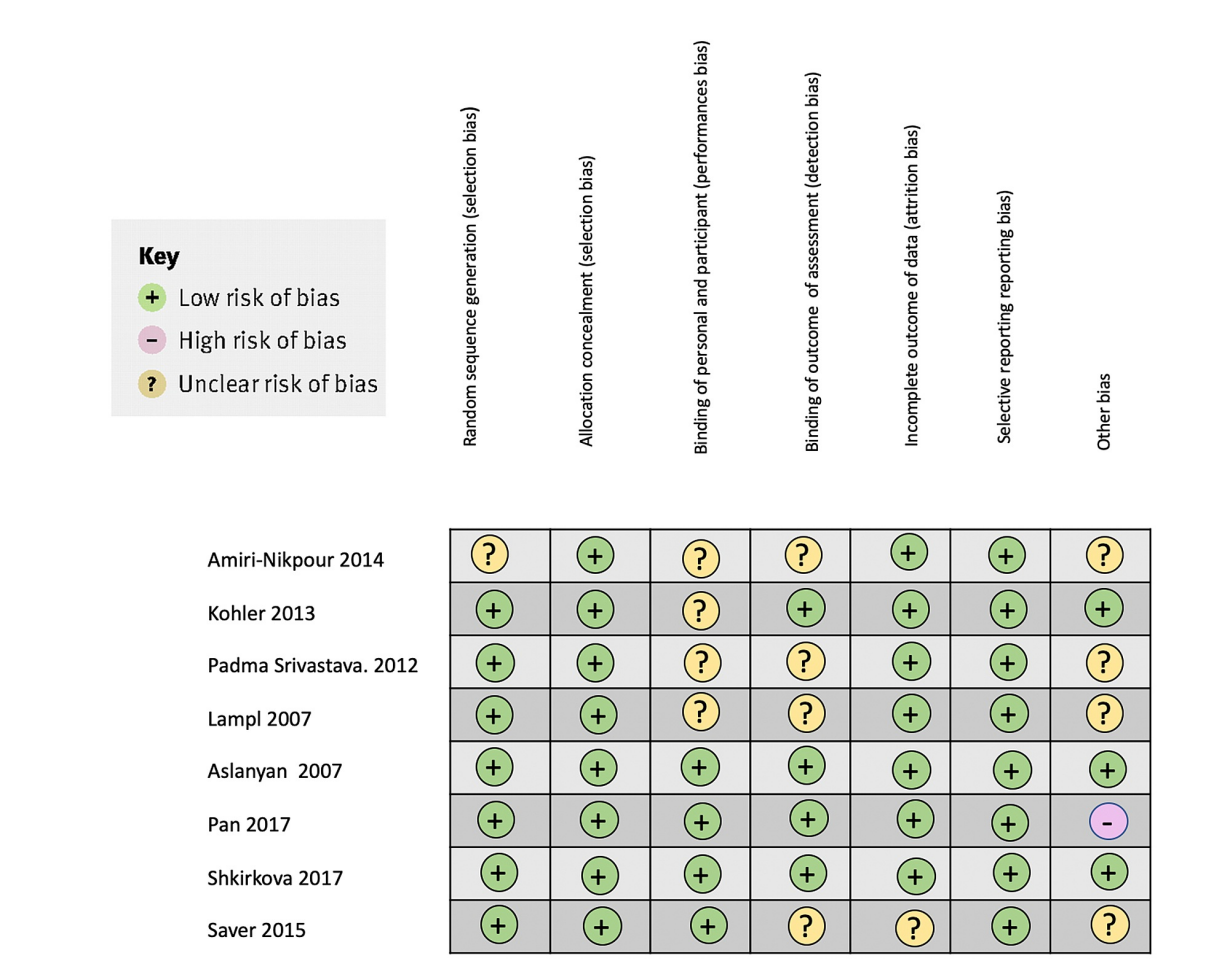
Bias of the clinical trials of minocycline and magnesium on ischemic stroke using the Cochrane collaboration's risk of bias tool

The study of Pan et al., where potassium-magnesium-enriched salt was introduced in the diet had a tiny sample. At the same time, the study's power was not good enough [6]. It is also mentioned that there was no pure magnesium group, and the benefits of the study could be due to synergistic effects of potassium and magnesium interaction and not just by the impact of magnesium itself [6]. Figure 2 summarizes the bias found in this study [2-6,10-13].

Discussion

Overall these clinical trials of minocycline have better results. The functional outcome improved in four of the five clinical trials [3,4,10,11]. While in the clinical trials with Mg, two out of four clinical trials had better outcomes [5,6]. Nevertheless, the clinical trials of Mg had study groups with more patients. The Mg trials were double-blinded, while minocycline trials were only blinded from the doctor's perspective. Minocycline's clinical trials lack a proper placebo; they indeed use a control group, but these control groups were not ideal because not all used a placebo.

The minocycline studies' main limitation was the sample size, lack of stratification, giving place to inexact findings. Further studies of minocycline should differentiate from IV or oral route administration. Simultaneously, sub-categorizing the infarcts could be crucial because some sub-type infarcts might have better minocycline outcomes than others [3,4,7,10,11]

Regarding the Mg clinical trials, the main problem was that these studies differed a lot from each other [5,6,12,13]. Ideally, future studies should investigate Mg administration with a shorter window of onset of symptoms.

Minocycline Clinical Trials: Results and Pathophysiology

Plasma levels of MMP-9 are increased in ischemic stroke. Increased levels of MMP-9 seem to be related to poorer outcomes and increased complications in ischemic stroke. The study by Switzer et al. measures the MMP-9 in patients after taking minocycline with and without tPA. The study found lower levels of MMP-9 at 72 hours in both tPA and non-tPA patients [10]. There are increased levels of MMP-9 after cerebral ischemia. When MMP-9 levels increase, there is a disruption of the brain-blood barrier. An increase in MMP-9 levels can increase by the use of tPA [19]. Minocycline decreased MPP-9 levels, leading to an improved outcome and decreased hemorrhagic conversion [10].

Amiri-Nikpour et al. with 60 patients, evaluated the National Institutes of Health Stroke Scale (NIHSS) scores after 90 days. Males had lower (NIHSS) scores at 30, 60 and 90 days. However, females did not show a better clinical outcome with the same parameter. Poly (ADP-ribose) polymerase-1 (PARP-1) activation promotes cell death. PARP-1 is an essential key mediator of minocycline [4]. Very interestingly, a study in mice showed that female mice did not benefit from minocycline when they had an ischemic stroke [20]. Previous studies have pointed out the ischemic death in females' brains seems to be triggered by the activation of caspases/cytochrome C instead of PARP-1 activation in men [21].

The study by Kohler et al. investigated if minocycline improves the modified ranking scale (mRS). While minocycline proved to be safe, it did not improve the functionality or the mortality, which differed from other clinical trials. The author thought it might be because of a type II error. The study was blinded on the investigator's side [7].

The study by Padma Srivastava et al. investigates the effects of oral minocycline on ischemic stroke. The clinical trial was a randomized, open-label, single-blinded study. This study had a control group with patients taking vitamin B as a placebo, unlike studies mentioned before with no placebo group. NIHSS and mRS scores were lower as compared to controls. Simultaneously, the modified Barthel Index (mBI) score was higher in patients with minocycline over placebo [3].

Finally, the Lampl et al. study was also an open-label, evaluator-blinded study with a proper control group. During the investigation, there were better functional outcomes as compared to placebo. Minocycline has many physiologic effects. Among the effects of minocycline are the following: inhibition of the activation of microglial cells, reduction in the migration of T-cells, suppression of free radical production, inhibition of metalloproteinases, inhibition of caspase I, III, cyclooxygenase 2, and decreased nitric oxide production [3,11]. Additionally, minocycline has been shown to have a protective effect on the spinal cord because it has reduced NMDA excitotoxicity [11].

Clinical Trials of Magnesium: Results and Pathophysiology

Previous clinical trials failed to show clinical improvement when Mg was administered in hospitalized patients in the ambulance within 12 hours of symptoms onset. Nevertheless, previous investigators thought it might benefit when Mg is given within two to three hours of symptom onset. Saver et al. discussed administering IV magnesium-sulfate within two hours of the onset of symptoms in pre-hospitalized patients. However, there was no clinical improvement at the end of the study [5].

An investigation by Shkirkova et al. studied the paramedic infusion of magnesium-sulfate within two hours of symptoms. This study's particularity was that the paramedics used a gravity-controlled tube with a fixed lumen that ensures more magnesium-sulfate delivery. The idea of this research was to reach more rapid target levels of magnesium in the body. Nevertheless, this innovative strategy did not improve clinical outcomes. Magnesium does not cross the BBB immediately. It takes four hours for the magnesium to peak in the cerebrospinal fluid (CSF). Magnesium-sulfate infusion with a gravity-controlled tube with a fixed lumen ensures more magnesium delivery. During the study, the levels of Mg increased more rapidly. Nevertheless, the study did not show clinical improvement in the participants [13]

Apart from the pre-hospital treatment with Mg, Pan et al. found that the patients with supplemental magnesium-potassium salt intake in their diets for six months improved their clinical outcomes. Mg may increase regional blood flow, enhance cellular metabolism recovery, and antagonize NMDA toxicity. Mg also seems to downregulate cyclooxygenase-2 expression and decreased glutamate toxicity in the hippocampal neurons [6].

A post hoc analysis by Aslanvan et al. showed that a subgroup of patients with lacunar stroke benefited from magnesium infusion in the pre-hospital setting [12]. Mg seems to have a more neuroprotective effect on the white matter over grey matter; because only a subgroup of patients with lacunar strokes benefitted from a pre-hospital infusion of Mg. It is essential to point out that lacunar infarcts affect white matter more than grey matter [12].

New Directions in the Clinical Trials of Stroke

In general, minocycline had better clinical outcomes than Mg trials. Minocycline and Mg seem to share some pathophysiological pathways regarding ischemic stroke [4,6]. Both drugs could have synergetic effects in the pre-hospital setting. It would be a great idea to combine these two drugs in future clinical trials. Minocycline and Mg are promising therapies for ischemic stroke in the pre-hospital setting. Because they are readily available, inexpensive, and generally safe, it was worth exploring these therapies' clinical trials [10].

## Conclusions

Minocycline and Mg are promising therapies for ischemic stroke in the pre-hospital setting. Because they are readily available, inexpensive, and generally safe, it was worth exploring these therapies through the systematic review of these clinical trials. Minocycline improves functional outcomes and disability scores. Minocycline decreases MMP-9 levels, which could reduce the hemorrhagic conversion of ischemic infarcts, independently using tPA. Minocycline might have a more significant effect on men than women because there are different molecular pathways of cerebral ischemia between both genres. PARP-1 activation seems to be the central mediator of cerebral ischemia in men, while caspases/cytochrome C appears to have a more influential role in women. 

Mg seems to have a more neuroprotective effect on the white matter over grey matter. So, Mg would be beneficial in patients with lacunar strokes. Supplementation of potassium-magnesium salt supplementation in the diet improved the functional outcomes of patients with ischemic strokes. Further studies could benefit from a gravity-controlled tube with a fixed lumen to ensure more Mg delivery. Because minocycline and Mg seem to share some pathophysiological pathways regarding ischemic stroke; both drugs could have a synergetic effect in the pre-hospital setting. Future studies will need more significant samples and a better selection of participants to increase the study's power and avoid selection bias, respectively. Further studies would benefit from the subcategorizing strokes and investigating the gender role concerning treatment. These directives will give a more robust conclusion regarding these drugs' neuroprotective effects on both therapies.
